# Suprapubic Lithotomy

**Published:** 1897-05

**Authors:** W. F. Chambers

**Affiliations:** Austin, Texas


For the Texas Medical Journal.

### SUPRAPUBIC LITHOTOMY.

BY W. F. CHAMBERS, M. D., AUSTIN, TEXAS.

[Read at Austin District Medical Society, March 19, 1897.]

IT WAS first performed by Franco about the middle of the sixteenth century. At that time it consisted in pushing the stone above the pubes by the fingers introduced into the rectum. It was practiced by first opening the membranous part of the urethra upon a catheter passed into the canal, through this incision a species of catheter, having spear-pointed stylet, was introduced into the bladder.

An incision was then made into the linea alba, above the symphysis pubis, of about four or five fingers' breadth, and the peritoneum detached to avoid wounding it.

The stylet was then pushed through the bladder, and used as a director for the knife, with which the bladder was divided anteriorly as far as the neck, and the stone extracted.

This was performed in England by Douglass, in 1719.

In performing the perineal operations, either lateral or median, there is great danger of injury to the urethra, also danger of rendering the patient impotent by injuring the seminal vesicles.

If the stone be very large, it cannot be extracted without first being crushed and washed out, all of which takes time, and any particles left are apt to form a nucleus for a new formation, and necessitate a second operation.

All of the work is done by the sense of touch alone, whereas, in the supra-pubic operation, you have the advantage of being able to see into the bladder, and also examine the prostate without further danger or mutilation of the patient.

The perineal operations are difficult to perform, bloody and dangerous, on account of narrow space in which the operator has to work.

In case of contracted bladder, there is great danger of the knife being pushed through the posterior wall, from which accident peritonitis would be sure to follow.

If the patient be fleshy, the distance from the skin to the inside of the bladder is too great for the operator to reach well with the finger.

In preparing the patient for the suprapubic operation, the rectum should be emptied, the pubes and perineum shaved, and the skin rendered thoroughly aseptic.

After being anæsthetized, the patient is placed on his back upon the table. The anterior surface of the bladder is covered by peritoneum as far down as the remains of the urachus, and posteriorly the peritoneum descends between the bladder and rectum. When these are empty, that portion of the bladder which is not covered by peritoneum, lies directly behind the pubic bone, but when the bladder is distended, it rises above the pubis and the peritoneum rolls back, and that portion of the bladder which is not covered by peritoneum, is exposed. In this case, the best to be done, is to inject about ten or twelve ounces of boric acid solution into the bladder.

It is not necessary to distend the rectum with a dilatable rubber bag, for it has been shown by actual measurement on cadaver that it does not elevate the fold of the peritoneum; it requires time for performance, and there is great danger of injuring the gut by overdistension, and the nerve distribution in the rectum is so extensive that it causes considerable rectal discomfort afterwards.

After distending the bladder, the catheter is allowed to remain as a guide. An incision is made above the pubis in the median line from two and a half to three inches in length. This is made directly in the linea alba, the aponeurosis of the external oblique being divided, the loose cellular tissue in front of the bladder is exposed. Great care should be taken in separating this cellular tissue in the median line with the handle of the scalpel, lest it should be torn free from the upper surface of the bladder, which would increase liability to urinary infiltration.

If the peritoneum now extends down into the wound, it can easily be pushed up out of the way, but with the bladder fully distended, it is very seldom seen. Now that the wall of the bladder has been fully exposed, hemorrhage should be controlled, and the bladder transfixed by a tenaculum at the upper portion of the abdominal wound, or stitched to the sides of the wound, so as to prevent collapse when the bladder is incised and the liquid allowed to escape. Some operators advise two stages in the operation in cases of septic cystitis. After the wall of the bladder has been exposed, the wound is dressed and the dressing allowed to remain for four or five days, when, if the wound has remained aseptic, it is covered with a layer of healthy granulations, which would greatly lessen the danger of urinary infiltration. At a second stage the bladder is opened and drained. When the incision into the bladder is made, the forefinger of the left hand is introduced, and the forceps passed into the bladder, with the finger as a guide, and the stone grasped and extracted.

Care should be taken that no fragments are left behind, and a careful examination made of the condition of the prostate, for often prostatic growths, obstructing the internal orifice of the urethra, act as a predisposing cause of calculus formation, and their removal also lessens liability of recurrence. If the bladder be in a healthy condition, it is deemed safe to completely suture the wound in the bladder at once. When this is done, Lembert's sutures should be used so as to bring in contact the serous coats of the two sides of the wound. When this is successfully done, the wound heals entirely in about three weeks.

The urine should be drawn every four hours to prevent over distension of the bladder.

If there is a complicating cystitis, and the bladder wall is much irritated, it is inadvisable to close the wound.

When the wound is left open, thorough and continuous drainage should be kept up for ten or twelve days, until the patient begins to pass healthy urine in considerable quantities through the urethra.

Cobb's method of drainage is effected by capillary attraction. It is obtained by a strip of iodoform gauze, one end of which passes into the bladder through a glass tube, and the other end, passing through a three-foot rubber tube, connects with a bottle over the bedside.

Dr. Wyeth uses a long rubber tube, which is made T shaped at one end. This end is introduced into the bladder, and its shape prevents it from slipping out; the other end connects with a bottle over the bedside, and the whole acts as a siphon to keep up continuous drainage.

The above described operation is easy and rapid to perform, and affords immediate relief. The patient recovers entirely in about four weeks.

The following is the history of a case that occurred in the practice Dr. John A. Wyeth:

R. B. G., age 36, contracted gonorrhœa in the spring of 1873, having had previous attacks, probably four, which had left him with a sub-acute catarrhal cystitis.

The acuteness of the attack of 1893 was soon ushered in with septic symptoms, chills and fever and sweats coming on about the third week. Emaciation was rapid, as well as the loss of sleep.

He visited celebrated mineral springs, noted for their diuretic properties, but only got worse.

He came to New York in July, 1893, and presented himself to Dr. Wyeth for treatment.

At this time the discharge had completely left him, but there was such a feeling of discomfort in the rectal, prostatic and vesical region that an exploration of the prostatic and vesical neck was deemed necessary. This was done by Dr. Wyeth under laughing gas.

I was instructed to go at once to my room and lie up for a day or two, but as I was feeling so well, I took a long walk, and on the following day found that I had no ability to void urine. The desire was frequent, attended with the most excruciating pain in the region of the kidneys, shooting down the spine, violent chills, high fever, nausea and vomiting.

The catheter did not relieve me. The vesical tenesmus became unbearable.

At 8 o'clock one evening I sent for Dr. Wyeth who, upon examination could find no enlargement of the prostate, and who, for reasons best known to surgeons, determined to do a supra pubic cystotomy that he might put the bladder at rest, as it was absolutely impossible to do an external urethrotomy, as the entire urethral tract was apparently in such a state of active inflammation, thus preventing the use of the sound subsequently had such an operation been done.

The relief from pain was immediate after the operation. On the third day the tube was removed from the bladder, as repeated attacks of hemmorrhage suggested this procedure. Convalescence was very slow. Mentally and physically I was a wreck for a long time. I gave up work and was put upon the liquor arsenici et auri bromidi in ten drop doses three times a day after meals, which was soon increased to fifteen drops three times a day. Under this treatment I rapidly improved. Up to that time I had not been able to empty the bladder perfectly. The urine was ammoniacal. The desire to micturate was frequent, but after eight weeks of the treatment I found myself greatly improved. Treatment was continued for three months, and to-day I am strong and well, vigorous and hearty; weigh 160 pounds, whereas formerly I never weighed more than 130.

